# Efficacy of carbonyl cyanide-3-chlorophenylhydrazone in combination with antibiotics against *Mycobacterium abscessus*

**DOI:** 10.1128/spectrum.01777-24

**Published:** 2024-12-27

**Authors:** Jifang Zheng, Qiang Liu, Haoran Wang, Fengmin Huo, Junnan Jia, Hairong Huang, Suting Chen

**Affiliations:** 1 National Clinical Laboratory on Tuberculosis, Beijing Key Laboratory for Drug-Resistant Tuberculosis Research, Beijing Chest Hospital, Beijing Tuberculosis and Thoracic Tumor Institute, Capital Medical University, Beijing, China; Seton Hall University, South Orange, New Jersey, USA

**Keywords:** CCCP, combination, *Mycobacterium abscessus*, antibiotic

## Abstract

Given the intrinsic resistance of *Mycobacterium abscessus* to a wide range of conventional antibiotics, it is urgent to explore new therapeutic approaches to manage this infection effectively. Carbonyl cyanide 3-chlorophenylhydrazone (CCCP), a proton pump inhibitor, has shown good bacteriostatic activity against *M. abscessus*. This study aimed to determine its synergistic antimicrobial effects when combined with commonly used antibiotics. The *M. abscessus* reference strain and 39 clinical isolates were collected. The minimum inhibitory concentration and fractional inhibitory concentration index were determined for the combined treatment using CCCP with various antibiotics including clarithromycin, amikacin, linezolid, bedaquiline, and clofazimine. A time-killing assay was used to measure the effect of the combined drug regimens quantitatively. The simultaneous use of CCCP with traditional antibiotics shows a synergistic effect across a wide range, significantly boosting the ability to inhibit the growth of *M. abscessus* throughout its growth phases. When CCCP is used in combination with clarithromycin, amikacin, and linezolid, it produces a synergistic effect on both the standard strain and most clinical isolates. When CCCP is paired with bedaquiline and clofazimine, it exhibits additive effects. Moreover, high levels of CCCP in combination with other antibiotics were found to rapidly eradicate the bacteria. The use of CCCP as a potential treatment for *M. abscessus* infections shows promising results, especially when combined with other antibiotics to achieve a potent bactericidal effect.

**IMPORTANCE:**

*Mycobacterium abscessus* poses a significant public health threat due to its intrinsic resistance to a broad spectrum of conventional antibiotics. This resistance necessitates urgently exploring novel therapeutic strategies to effectively combat infections caused by this pathogen. Our previous research has identified carbonyl cyanide-3-chlorophenylhydrazone (CCCP) as a potent direct antimicrobial agent against *M. abscessus* and as an enhancer of clarithromycin activity. Our results demonstrate that the concurrent administration of CCCP with traditional antibiotics exhibits a synergistic effect across a wide range, which could be crucial for overcoming the challenges posed by *M. abscessus* infections. Furthermore, the use of high concentrations of CCCP in combination with other antibiotics was found to rapidly eliminate *M. abscessus*, suggesting a potential therapeutic advantage. These insights not only advance our understanding of antimicrobial synergy but also hold promise for the development of more effective treatment regimens against drug-resistant *M. abscessus* infections.

## INTRODUCTION

In recent years, there has been a significant increase in caused by non-tuberculous mycobacteria (NTM), with *Mycobacterium abscessus* infections being among the most common (1). *M. abscessus* is a rapidly growing mycobacterium that can be differentiated into three subspecies—*abscessus*, *bolletii*, and *massiliense*—based on genomic sequencing, with the subspecies *abscessus* being the most commonly identified in clinical settings (2). *M. abscessus* mainly causes infections in the lungs and soft tissues of the skin (3). Effective management of infections caused by this bacterium usually involves a customized combination of multiple drugs. This treatment approach is based on *in vitro* susceptibility testing and guided by clinical expertise. The current treatment guidelines recommend using macrolides, beta lactams (such as cefotaxime, imipenem, or meropenem), and aminoglycosides in treatment plans (4). Due to its inherent resistance to conventional antibiotics, the cure rate for this infection is less than 60% (5). The treatment success rate is relatively high (54%) for the subspecies *massiliense* and relatively low (34%) for the subspecies *abscessus* (6). Therefore, there is an urgent need to develop innovative antimicrobial agents and explore alternative treatment strategies to effectively combat infections caused by *M. abscessus*. Pursuing new therapeutic options is essential to improve treatment outcomes and address the growing challenge of drug resistance in managing these infections.

Carbonyl cyanide 3-chlorophenylhydrazone (CCCP) is a proton pump inhibitor that disrupts the proton motive force, essential for inhibiting efflux pumps' functioning. Efflux pumps are a widespread mechanism that leads to antibiotic resistance in bacteria (7). Although CCCP has primarily been used in research to study bacterial efflux and biofilm formation, our earlier studies have shown that it has a direct strong antimicrobial effect on *M. abscessus* and enhances the activity of clarithromycin (8). Based on these findings, the current study aims to evaluate the combined antimicrobial effectiveness of CCCP with three frequently used and two new antibiotics: clarithromycin (CLA), amikacin (AMK), linezolid (LZD), bedaquiline (BDQ), and clofazimine (CFZ) and explore the potential of using CCCP in clinical combination therapies to improve treatment outcomes for infections caused by this challenging pathogen.

## MATERIALS AND METHODS

### Bacterial culture

The reference strain of *M. abscessus* (ATCC 19977) and 39 isolates were kept in the biobank of Beijing Chest Hospital of Capital Medical University. All *M. abscessus* isolates were identified into subspecies by multi-locus sequence analysis and comparison, including *hsp65*, *rpoB,* and *erm(41*) gene. All these strains were inoculated on Lowenstein-Jensen (L-J) medium and cultured for 3–7 days. The growth phenotype of the clinical strains on the medium was documented.

### Minimum inhibitory concentration measurement

CLA, AMK, LZD, BDQ, and CFZ were purchased from MCE (Shanghai, China), and CCCP was purchased from Solarbio (Beijing, China). Drug susceptibility testing was performed according to the guidelines of the Clinical and Laboratory Standards Institute (CLSI) (referred to as drug susceptibility testing) (9). The antibiotics were diluted using Mueller-Hinton (MH) broth medium, and the concentration range of CCCP was 0.03–16.00 mg/L, CLA was 0.01–2.00 mg/L, AMK was 0.25–128.00 mg/L, LZD was 0.25–128.00 mg/L, BDQ was 0.01–2.00 mg/L, and CFZ was 0.01–8.00 mg/L. The colonies were scraped from the slant of the L-J medium and prepared into a fresh bacterial solution with a concentration of 0.5 McFarland standard. The solution was diluted at 1:200 and then inoculated into 96-well microtiter plates. After incubation at 37°C for 72 h, a mixture containing 20 µL of Alamar Blue and 50 µL of 5% Tween 80 was added to each well, and the bacterial growth was assessed after incubation at 37°C for 24 h. The lowest drug concentration that prevented the liquid from changing from blue to pink was determined as the 1MIC value, and all experiments were repeated three times.

### Determination of fractional inhibitory concentration index

The combination drug sensitivity test was performed using the checkerboard dilution method, and the concentration range of the drugs used in the combination drug sensitivity test was determined according to the MIC results of each single drug (10). CCCP, CLA, AMK, LZD, BDQ, and CFZ were multiply diluted so that the working concentrations were 8MIC, 4MIC, 2MIC, 1MIC, 1/2MIC, 1/4MIC, 1/8MIC, and 1/16MIC, respectively. After incubation at 37°C for 72 h, a mixture of 20 µL of Alamar Blue and 50 µL of 5% Tween 80 was added to each well, and the bacterial growth was assessed after incubation at 37°C for 24 h. The MIC values of the two drugs were recorded, and the FICI was calculated to determine the effect of the combination of the two drugs. FICI was calculated as follows: FICI = MIC of drug A in combination/MIC of drug A alone + MIC of drug B in combination/MIC of drug B alone. FICI ≤ 0.5 indicates synergy, 0.5 < FICI ≤ 1 indicates additivity, 1 < FICI ≤ 2 indicates indifference, and FICI > 2 indicates antagonism.

### Time-killing assay

Time-killing experiments were performed on the *M. abscessus* reference strain (11). For bacteriostatic effect, the strain was grown logarithmically in MH broth, prepared into a fresh bacterial solution with a concentration of 0.5 McFarland standard, and diluted 200 times. The low-concentration (0.25MIC and 0.5MIC) antibiotic combinations were added and further incubated at 37°C for another several days. At the 0, 2, 4, and 6 day time points, 100 µL of the suspension was diluted into 10 times gradient. Then, 10 µL aliquots was spotted on MH agar plates and counted after incubation at 37°C for at least 3 days, and time-killing curves were plotted to evaluate the bacteriostatic effect of the drugs alone and in combination. For the bactericidal effect, the strain grew logarithmically and adjusted into OD = 0.1. The high-concentration (4MIC and 10MIC) antibiotic combinations were added and further incubated at 37°C. At the 0, 1, 2, 3, and 7 day time points, 100 µL of the suspension was diluted into 10 times gradient. Colony counting was done only on plates with 30–300 colonies. The bactericidal activity, which is defined as the killing of 99.9% of the initial bacteria, can be determined from time-killing curves by observing whether there is a ≥3 log10 decrease in CFU/mL. Synergy is defined as a ≥2 log10 decrease in CFU/mL between the combination of substances and its most effective individual constituent (12).

### Statistical analysis

SPSS 24.0 software and Graph Pad Prism 8.0 software were used for data analysis. Non-paired *t*-test was used to determine the significance of the differences between groups in the antimicrobial susceptibility determination. Differences were considered statistically significant at *P* < 0.05.

## RESULTS

### Synergistic bactericidal effects of CCCP in combination with antibiotics

**Fig 1:**
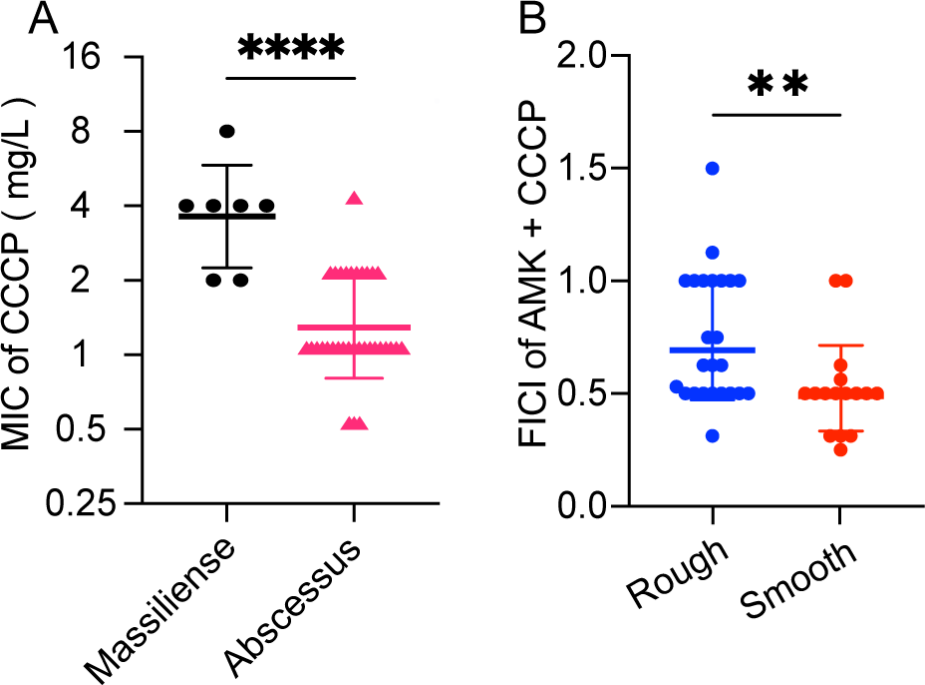
The bacteriostatic effect of CCCP against *M. abscessus*. (A) The MIC distribution of CCCP against subspecies *abscessus* and subspecies *m*assiliense. (B) The FICI distribution of amikacin (AMK) plus CCCP in different morphotypes *M. abscessus*. The horizontal line in the figure represents the geometric mean, while the upper and lower lines, respectively, represent the lower and upper limits of the 95% confidence interval. The data underwent a twofold logarithmic transformation, followed by a non-paired *t*-test to assess group differences, with variance homogeneity confirmed by a *P* > 0.05,**P < 0.01, ****P < 0.0001.

A total of 32 strains of *M. abscessus* subsp. *abscessus* and 7 strains of *M. abscessus* subsp. *massiliense* were identified. First, the minimum inhibitory concentration (MIC) of each studied drug against the clinical isolates was determined (see Tables 1 and 2). The MIC of CCCP was significantly higher in subsp. *massiliense* than that in subsp. *abscessus* (Fig. 1A). Following this, the most synergistic combinations were evaluated using the FICI as previously described (see Tables 1 and 3). For the *M. abscessus* reference strain, the FICI for the CCCP/CLA combination was 0.31, CCCP/AMK was 0.31, CCCP/LZD was 0.56, CCCP/BDQ was 1, and CCCP/CFZ was 0.56. Among the 39 clinical isolates, the CCCP/CLA combination exhibited synergistic effects in 22 strains (56.4%), additive effects in 16 strains (41.0%), and no effect in 1 strain (2.6%). The CCCP/AMK combination showed synergistic effects in 20 strains (51.3%), additive effects in 17 strains (43.6%), and no effect in 2 strains (5.1%). The CCCP/LZD combination had synergistic effects in 23 strains (59.0%) and additive effects in 16 strains (41.0%). The CCCP/BDQ combination resulted in synergistic effects in 1 strain (2.6%), additive effects in 25 strains (61.5%), and no effect in 14 strains (35.9%). For the CCCP/CFZ combination, there was a synergistic effect in 1 strain (2.6%), additive effects in 33 strains (82.0%), and no effect in 6 strains (15.4%). Notably, within the CCCP/AMK group, the FICI values for the smooth *M. abscessus* were slightly lower than those for the rough variant (Fig. 1B). There was no significant difference in other combinations between the isolates with rough and smooth morphotypes (data not shown).

**TABLE 1:** MIC and FIC values of each antimicrobial agents in *M. abscessus* clinical isolates

Strain no.	Colony morphology	MIC (mg/L)						FIC (combined with CCCP)				
		CCCP	CLA	AMK	LZD	BDQ	CFZ	CLA	AMK	LZD	BDQ	CFZ
Subspecies *abscessus*												
1	Smooth	1.00	0.13	2.00	4.00	0.63	0.50	0.75	0.63	0.25	0.50	0.63
2	Rough	2.00	1.00	2.00	8.00	0.13	0.50	0.38	0.63	0.75	1.00	0.75
3	Smooth	1.00	0.06	2.00	8.00	0.06	0.50	0.38	0.75	0.38	2.00	1.00
4	Rough	0.50	0.06	2.00	2.00	0.13	0.25	0.31	1.00	0.50	1.00	0.75
5	Smooth	1.00	0.03	1.00	4.00	0.13	0.25	0.50	1.00	1.00	1.00	2.00
6	Smooth	2.00	0.50	4.00	4.00	0.06	0.25	0.50	0.50	0.50	2.00	0.75
7	Smooth	2.00	0.50	2.00	8.00	0.06	0.25	0.50	0.50	0.38	0.63	0.50
8	Rough	1.00	0.25	2.00	4.00	0.06	0.25	0.50	0.50	0.50	0.63	0.63
9	Smooth	1.00	0.25	2.00	4.00	0.06	0.25	1.00	0.63	0.75	1.50	0.75
10	Rough	1.00	0.13	4.00	2.00	0.06	0.25	0.75	0.31	1.00	1.50	0.75
11	Rough	2.00	0.13	2.00	8.00	0.06	0.25	1.00	0.50	0.50	1.00	1.00
12	Smooth	2.00	0.13	2.00	4.00	0.06	0.25	0.56	0.53	0.56	1.00	0.63
13	Smooth	2.00	0.13	1.00	16.00	0.06	0.25	0.63	1.13	1.00	1.00	1.00
14	Smooth	1.00	0.06	2.00	8.00	0.06	0.25	0.38	0.63	0.75	1.00	0.75
15	Rough	1.00	0.06	2.00	2.00	0.06	0.25	0.50	0.50	0.37	1.50	0.56
16	Smooth	1.00	0.03	4.00	4.00	0.06	0.25	0.31	1.00	0.50	1.00	1.00
17	Smooth	1.00	0.02	4.00	4.00	0.06	0.25	0.38	1.00	1.00	1.50	1.00
18	Rough	1.00	0.50	4.00	4.00	0.03	0.25	0.75	0.50	0.31	0.63	0.75
19	Smooth	2.00	0.06	4.00	4.00	0.03	0.25	0.75	1.00	0.75	1.50	1.00
20	Rough	4.00	0.06	2.00	8.00	0.03	0.25	0.50	0.50	0.50	2.00	0.63
21	Rough	1.00	0.06	2.00	4.00	0.03	0.25	0.31	0.25	1.00	1.00	1.00
22	Smooth	1.00	0.03	4.00	4.00	0.03	0.25	1.00	1.00	0.50	2.00	1.00
23	Smooth	2.00	0.03	2.00	8.00	0.03	0.25	0.50	0.50	0.50	2.00	0.63
24	Rough	2.00	0.02	4.00	4.00	0.03	0.25	0.50	0.50	0.50	1.50	1.00
25	Smooth	2.00	0.02	4.00	4.00	0.03	0.25	0.56	0.50	0.50	1.00	0.75
26	Rough	1.00	0.02	4.00	4.00	0.03	0.25	0.50	0.31	0.38	1.50	1.50
27	Rough	1.00	0.02	4.00	4.00	0.03	0.25	0.31	0.50	0.37	1.00	0.75
28	Smooth	1.00	0.02	1.00	4.00	0.03	0.25	0.50	0.50	1.00	1.00	1.00
29	Smooth	1.00	0.01	2.00	4.00	0.03	0.25	1.00	1.00	1.00	0.75	0.63
30	Rough	1.00	0.25	2.00	4.00	0.13	0.13	0.56	0.56	0.75	1.50	0.75
31	Smooth	0.50	0.02	1.00	2.00	0.06	0.13	0.50	1.00	0.50	0.75	2.00
32	Smooth	0.50	0.02	4.00	4.00	0.03	0.13	0.50	0.50	1.00	1.25	0.63
Subspecies *massiliense*												
33	Smooth	8.00	0.03	4.00	8.00	0.06	0.50	0.50	0.50	0.50	1.00	1.00
34	Smooth	4.00	0.02	2.00	4.00	0.13	0.25	0.38	1.50	0.50	1.00	0.75
35	Rough	2.00	0.01	1.00	4.00	0.13	0.25	0.50	0.50	0.50	0.75	1.50
36	Smooth	4.00	0.06	4.00	16.00	0.06	0.25	0.63	0.31	0.75	1.00	1.00
37	Smooth	4.00	0.06	4.00	8.00	0.03	0.25	0.75	0.50	0.50	1.00	1.50
38	Smooth	4.00	0.06	4.00	8.00	0.03	0.25	0.63	0.75	0.75	1.00	1.25
39	Rough	2.00	0.02	4.00	4.00	0.03	0.25	1.50	1.00	0.25	0.75	0.75

**TABLE 2:** The distribution of the MIC values of each drug in *M. abscessus* clinical isolates

Drug	MIC range (mg/L)	MIC_50_ (mg/L)	MIC_90_ (mg/L)
CCCP	0.50–8.00	1.00	4.00
CLA	0.01–1.00	0.06	0.50
AMK	1.00–4.00	2.00	4.00
LZD	2.00–16.00	4.00	8.00
BDQ	0.03–0.13	0.06	0.13
CFZ	0.13–0.50	0.25	0.50

**TABLE 3:** The synergictive effect of CCCP and other drugs against the clinical isolates of *M. abscessus*

Drug	Synergy (*n*, %)	Additive (*n*, %)	Indifference (*n*, %)
CLA	22 (56.41%)	16 (41.03%)	1 (2.56%)
AMK	20 (51.28%)	17 (43.59%)	2 (5.13%)
LZD	23 (58.97%)	16 (41.03%)	0 (0)
BDQ	1 (2.56%)	24 (61.54%)	14 (35.90%)
CFZ	1 (2.56%)	32 (82.05%)	6 (15.39%)

**Fig 2:**
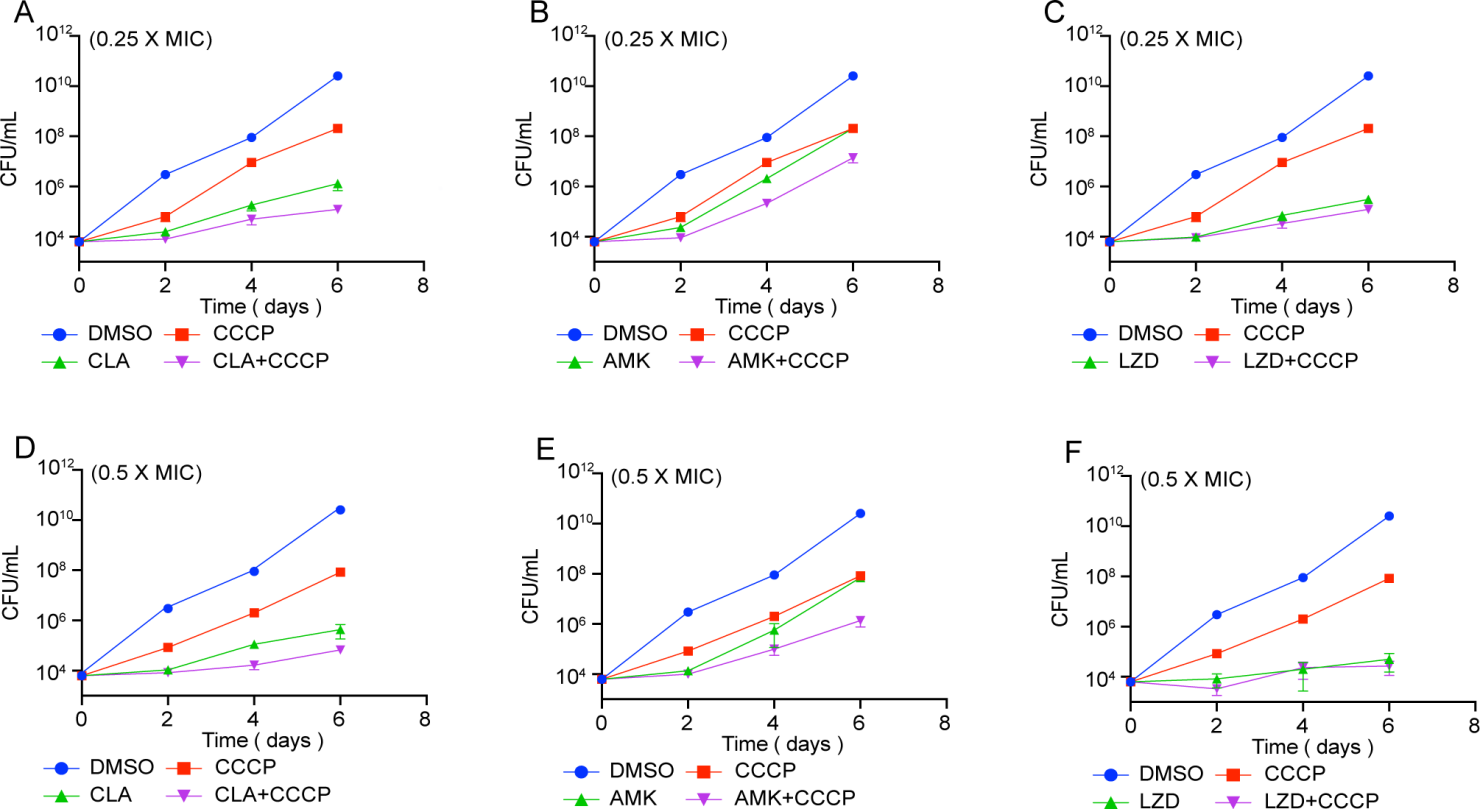
Bacteriostatic effect of *M. abscessus* following treatment with low concentrations (0.25MIC or 0.5MIC) of clarithromycin (CLA), AMK, or linezolid (LZD) plus CCCP. The experiment was carried out with three replications.

The time-killing curves for the reference strain treated with single drugs and two drugs in combination showed that combining CCCP with antibiotics had a potent bacteriostatic effect (Fig. 2). Combining 0.5MIC CCCP with 0.5MIC of CLA and AMK, and 0.25MIC CCCP with 0.25MIC of CLA and AMK resulted in a decrease of bacterial count by at least 1.00 log10 CFU/mL after 6 days of antibiotic incubation compared to the untreated group or single drug-treated group. When combined with LZD, the bacterial count decreased by only 0.27 log10 CFU/mL (0.5MIC) and 0.39 log10 CFU/mL (0.25MIC) compared to LZD alone. These results indicated that combining CCCP with CLA and AMK had a better bacteriostatic effect, while the effect was less pronounced when combined with LZD.

### Rapid bactericidal effect of high-concentration CCCP combination therapy against *M. abscessus*

**Fig 3:**
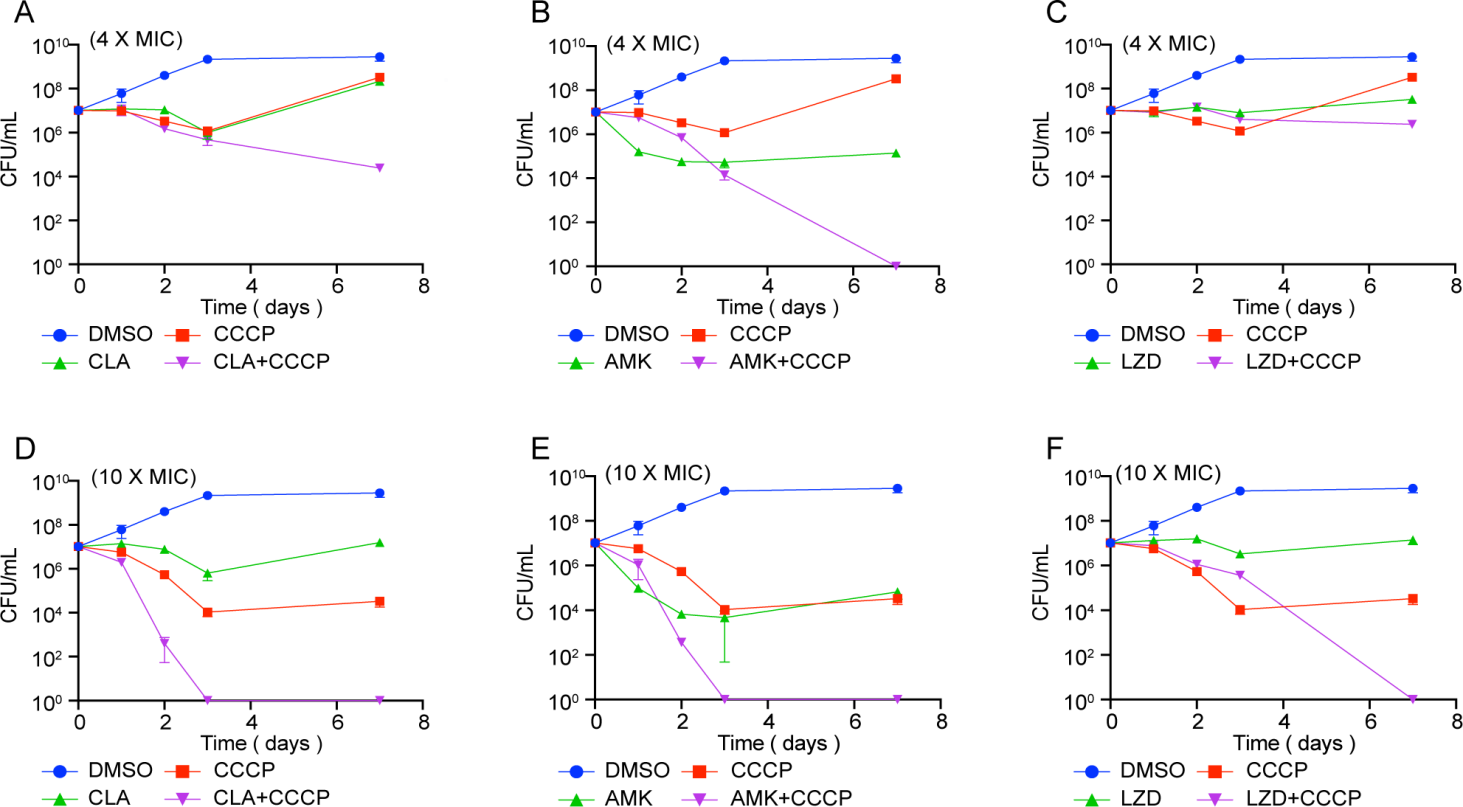
Bactericidal effect *M. abscessus* following treatment with high concentrations (4MIC or 10MIC) of CLA, AMK, or LZD plus CCCP. The experiment was carried out with three replications.

The bacteria were exposed to high concentrations (4MIC and 10MIC) of drug combinations for 7 days (Fig. 3). It was found that the combination of 4MIC CCCP with 4MIC antibiotics demonstrated superior bactericidal effects compared to the use of 10MIC antibiotics alone. Additionally, the 10MIC of each antibiotic combined with 10MIC CCCP completely cleared the bacteria within 7 days. In comparison to using either CCCP or antibiotics alone, the combined therapeutic approach resulted in a significant reduction of more than 3 log10 in the CFU by day 7. Notably, CCCP significantly enhanced the bactericidal action of AMK, with only 4MIC of each antibiotic sufficient to completely eradicate the bacteria within 7 days. For CLA and LZD, a higher concentration of antibiotics was required to achieve complete bacterial clearance, and a higher concentration of CCCP in combination was also necessary.

### Bactericidal effects of combination therapy with CCCP against *M. abscessus* at different growth phases

**Fig 4:**
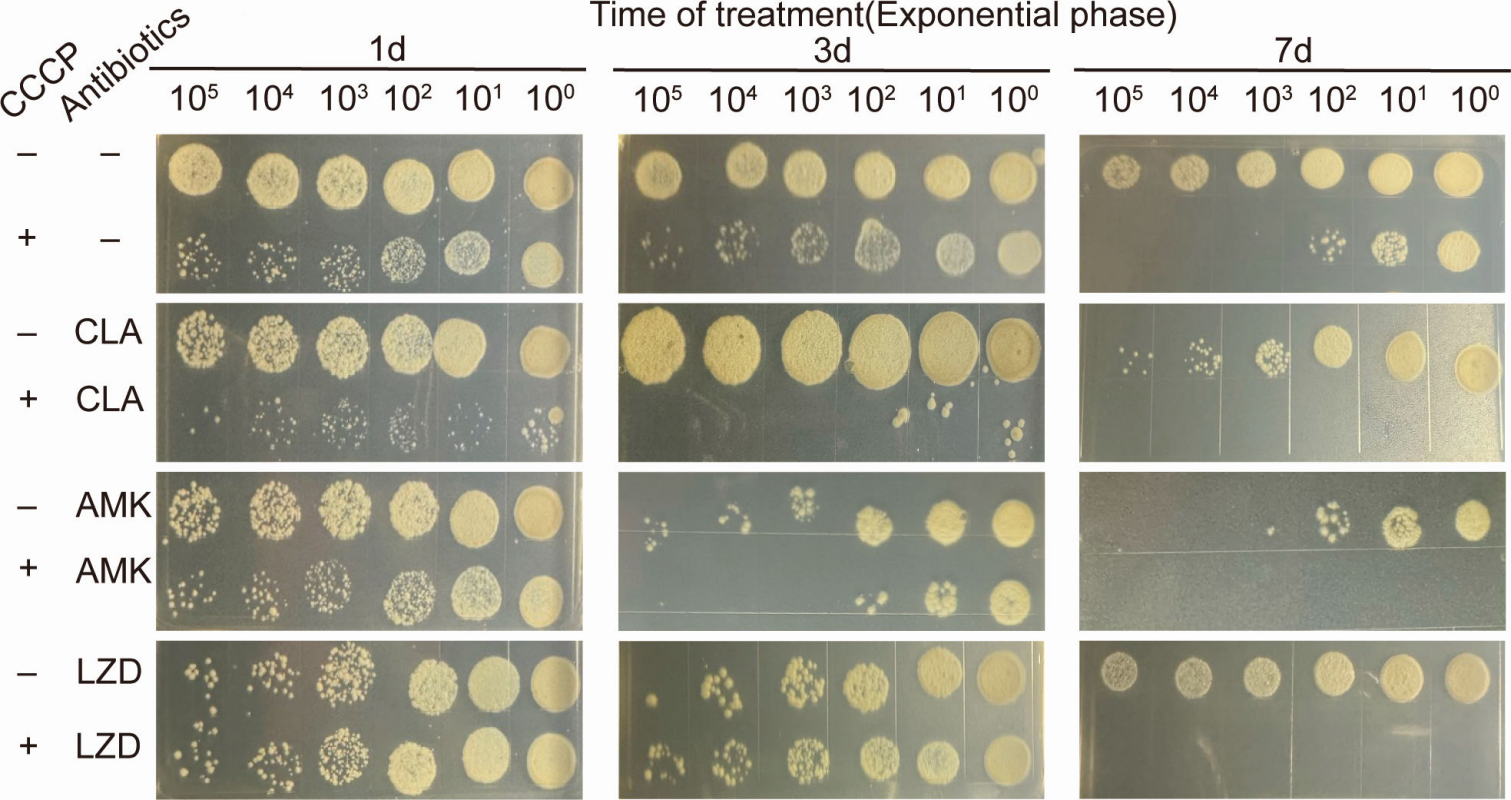
Survival of *M. abscessus* in exponential-phase (3 days after inoculation) after the addition of 0.625 mg/L CLA, 20 mg/L AMK, or 40 mg/L LZD in the absence or presence of 10 mg/L CCCP and followed by further agitation for 1, 3, or 7 days. One of three representative experiments was shown.

**Fig 5:**
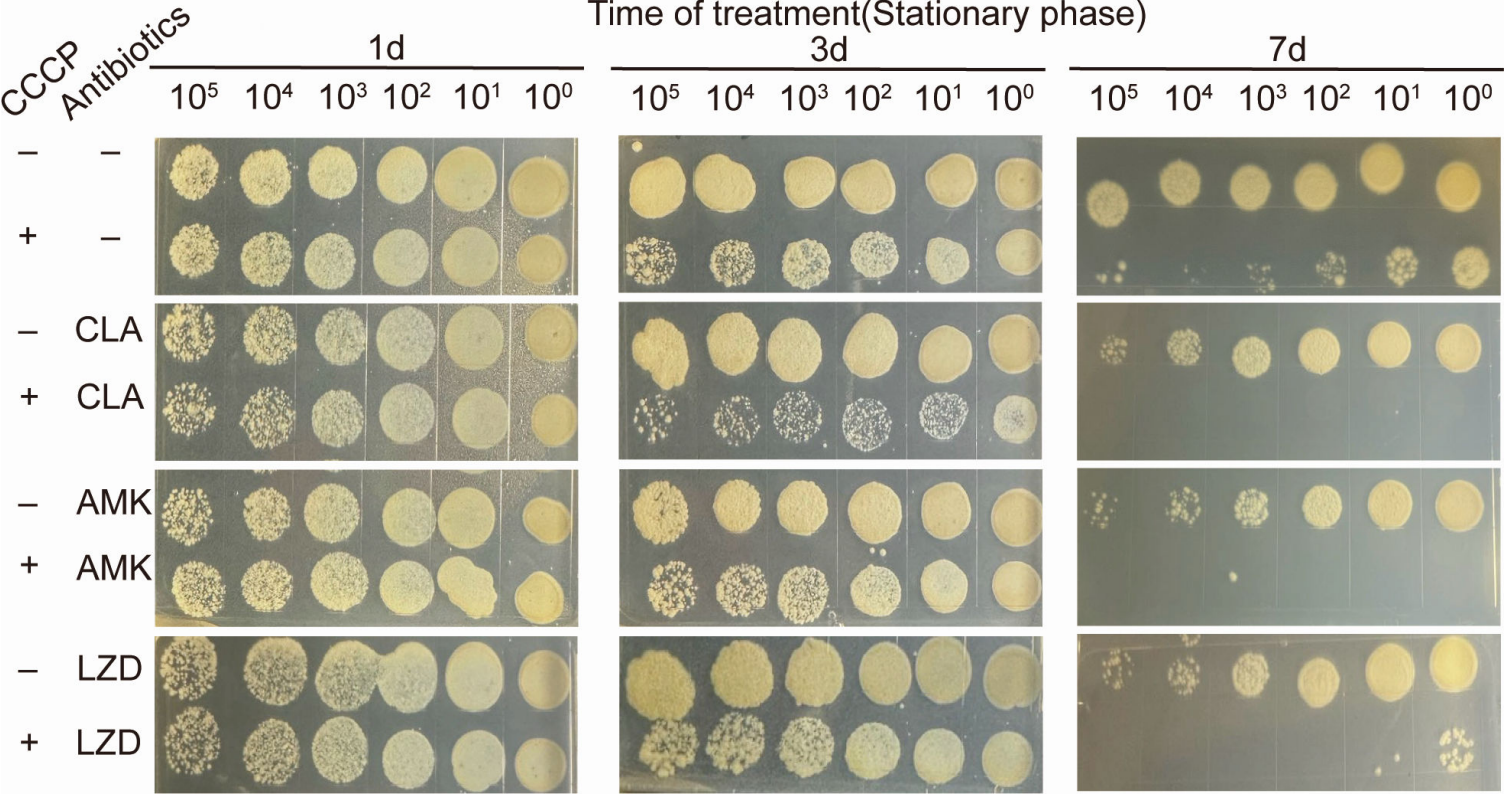
Survival of *M. abscessus* in stationary-phase (7 days after inoculation) after the addition of 0.625 mg/L CLA, 20 mg/L AMK, or 40 mg/L LZD in the absence or presence of 10 mg/L CCCP, followed by further agitation for 1, 3, or 7 days. One of three representative experiments was shown.

**Fig 6:**
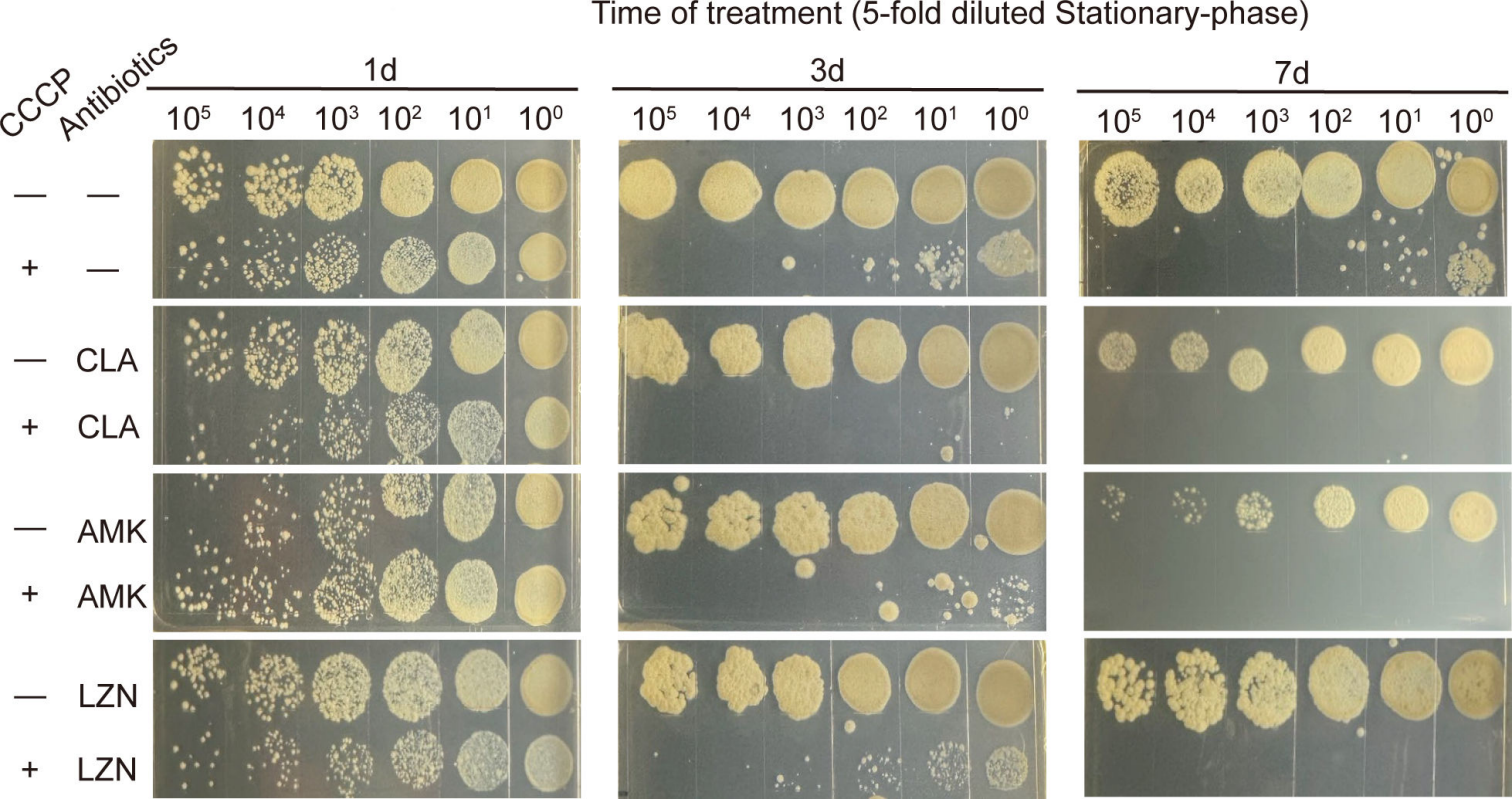
Survival of fivefold diluted stationary-phase *M. abscessus* (7 days after inoculation) atter the addition of 0.625 mg/L CLA, 20 mg/L AMK, or 40 mg/L LZD in the absence or presence of 10 mg/L CCCP, followed by further agitation for 1, 3, or 7 days. One of three representative experiments was shown.

Research has shown that the lethal effect of CCCP on aminoglycosides is correlated with the growth state of bacteria (13). To determine if the synergistic effect of CCCP to kill *M. abscessus* is tied to the different stages of growth, the reference strain was cultured to both the logarithmic phase (Fig. 4) and the stationary phases (Fig. 5) before being treated with a drug concentration of 10MIC for 7 days. The results showed that the combination therapy effectively eradicated the bacteria in both growth phases, performing significantly better than monotherapy. Furthermore, it was noticed that the survival rate of bacteria in the stationary phase was slightly higher than that in the logarithmic phase. In light of this, we investigate the impact of cell density by diluting the stationary phase bacteria five times before drug treatment. We found that CCCP could significantly enhance the ability of antibiotics to prevent the growth of bacteria in the stationary phase and that the number of bacteria also affects the effectiveness of the treatment (Fig. 6).

## DISCUSSION

The incidence of *M. abscessus* infections is high and continues to increase each year. Treatment success rates currently range from only 25% to 58% (14). Current therapeutic strategies for *M. abscessus* infections primarily rely on drug combinations with macrolide as the core drug (15). However, the emergence of resistance to CLA has limited effective treatment options for this condition (16). Recent research has focused on the correlation between drug efflux pump systems and antibiotic resistance, revealing that efflux pump inhibitors can reduce bacterial resistance levels and provide a new approach to addressing antimicrobial resistance (17). Our preliminary research has shown that the efflux pump inhibitor CCCP has strong antimycobacterial activity against *M. abscessus* and can lower the MIC of the bacterium for CLA and levofloxacin (10, 18). As a result, our current study further evaluated the *in vitro* antimicrobial activity of CCCP in combination with CLA, AMK, LZD, BDQ, and CFZ against *M. abscessus*.

The result of the checkerboard synergy test showed that when CCCP was combined with each of these five drugs, it had good synergistic or additive effects, especially on the smooth strains. Time-killing assays were conducted with three combinations (CCCP with CLA, AMK, and LZD), and the results indicated that CCCP combined with CLA, AMK, and LZD had good bacteriostatic activity, especially when CCCP was combined with AMK. AMK, as an aminoglycoside antibiotic, is taken up by bacteria mainly through the proton motive force (PMF, ΔμH^+^) (19). Studies have shown that efflux pump inhibitors, which use PMF to expel antibiotics from the cell, can act as adjuvants for aminoglycosides (20). It has been found that certain protonated groups (such as CCCP and FCCP) can enhance the effects of aminoglycosides, increasing the levels of hydroxyl radicals within bacterial cells and blocking the bactericidal action of antibiotics against exponential-phase bacteria (13).

CCCP has been found to inhibit the bactericidal effects of antibiotics, such as β-lactams and fluoroquinolones, against exponential-phase *Escherichia coli* by suppressing ATP synthesis and cell growth (13). However, in our current study, combining CCCP with antibiotics proved effective against *M. abscessus* at various growth stages. Higher concentrations of CCCP (10MIC) in combination with different drugs completely cleared the bacteria within a week. Previous research has suggested that when the concentration of CCCP is 12.5 µg/mL or higher, the THP-1 cell survival rate drops below 80% (10). In our experiment, the highest concentration of CCCP used was 10 µg/mL, so further cytotoxicity experiments should determine if this concentration is safe for different cells.

In addition, our experiments showed that using 10MIC CCCP killed *M. abscessus*, better than using CLA, AMK, and LZD at equivalent concentrations. Both 4MIC and 10MIC CCCP treatments greatly lowered the number of bacteria in the initial 3 days. But after 7 days, the bacteria increased again in the 4MIC group, while the 10MIC group kept the bacteria low, like on day 3. Thus, we proposed two hypotheses about this phenomenon. First, CCCP might not work as well over time. CCCP breaks down in high-temperature condition, so it does not kill bacteria as well and the bacteria grow faster. This might explain why the bacteria increased later on. Second, low amounts of CCCP induced resistance in *M. abscessus*. We hypothesized that at low concentrations, CCCP may not completely disrupt the intracellular proton gradient, leading to changes in intracellular ATP concentrations. These changes might encourage the formation of protein aggregate bodies within bacterial cells, pushing the bacteria into a state of deep dormancy stage called the persister state (21). In this state, bacteria are less susceptible to antibiotics as their metabolic activities slow down (22). However, at high concentrations, CCCP might quickly disrupt the cell membrane’s integrity, depleting intracellular ATP and hindering the cell’s ability to maintain normal physiological functions (23). This rapid disruption may prevent the formation of protein aggregate bodies, or if they do form, they cannot stabilize due to the severe stress on the cell. Therefore, under the influence of high concentrations of CCCP, *M. abscessus* may not enter a deep dormancy state, thus maintaining susceptibility to antibiotics. Furthermore, at high concentrations, CCCP may directly interact with bacterial target proteins, disrupting their normal functions, rather than indirectly affecting drug resistance by influencing ATP concentrations (24). This direct interaction may lead to the rapid death of bacteria, leaving no opportunity for the development of resistance.

This study has some limitations. First, the antimicrobial activities determined *in vitro* may not necessarily reflect *in vivo* effectiveness, especially for synergistic effects, which need to be confirmed through animal models or human trials. Second, the limited number of clinical strains selected from a single institution can lead to sample bias, thus requiring a larger sample size for further validation in future studies. Third, CCCP interferes with mitochondrial depolarization and induces the process of mitochondrial autophagy, so the use of CCCP in humans should be approached with extreme caution.

This study demonstrated that CCCP can improve the bactericidal effects of various antimicrobial drugs against *M. abscessus*. The decrease in MIC due to the synergistic effects could lead to better outcomes and enhanced clinical efficacy, reducing the development of drug resistance in *M. abscessus*. In the future, small-molecule drugs derived from CCCP may have promising applications.

## Data Availability

The raw data supporting the conclusions of this article will be made available by the authors without undue reservation.
